# Stabilization of [(N_5_)_2_BX]^2–^ and [(N_5_)_2_B_2_X_2_]^2–^ (X = H, F, Cl, Br) by Conjugation and Hyperconjugation Effects

**DOI:** 10.1021/acs.inorgchem.4c04865

**Published:** 2025-01-27

**Authors:** Dongyi Xiao, Qianyue Yu, Haifeng Yi, Yan Zhang, Gregory H. Robinson, Henry F. Schaefer

**Affiliations:** † College of Life and Environmental Sciences, Minzu University of China, Beijing 100081, China; ‡ Center for Computational Quantum Chemistry, University of Georgia, Athens, Georgia 30602, United States

## Abstract

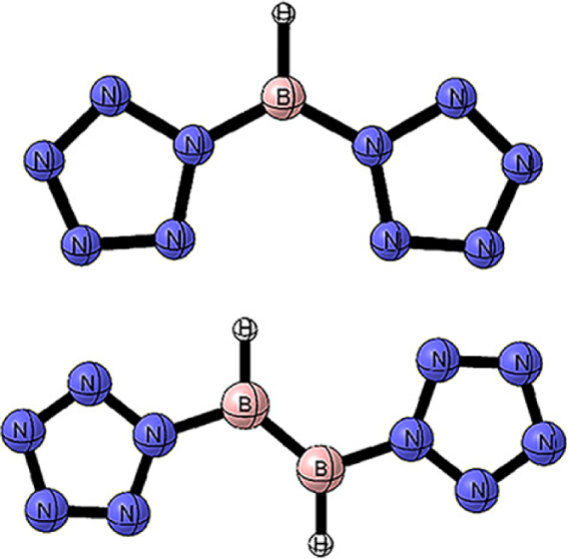

The isolation of nucleophilic boron bases has led to a paradigm shift in boron chemistry. Previous studies of the bis(carbene) borylene complexes revealed that these compounds possess strong donor abilities, and their reaction inertness is due to the large steric hindrance between boron reagents and reactant. In the present study, we have theoretically studied the [(N_5_)_2_BX]^2–^ and [(N_5_)_2_B_2_X_2_]^2–^ compounds (X = H, F, Cl, Br). Their electronic structures and properties are discussed by using the NBO, LOL, and ELF methods. We found that both π-conjugation and hyperconjugation effects can effectively stabilize the substituted nucleophilic anionic boron compounds [(N_5_)_2_BX]^2–^ and [(N_5_)_2_B_2_X_2_]^2–^. Substituents, especially X = H, stabilize the boron center through highly delocalized π-bonding, involving the formally “empty” in-plane *p* orbitals of the boron atom. While the halogen substituents have high electron withdrawal ability, leading to systems being less stable, we suggest the borinium anions [(N_5_)_2_BH]^2–^ and [(N_5_)_2_B_2_H_2_]^2–^ as possible synthetic targets of novel environmentally friendly catalysts.

## Abstract 2

Among the eight compounds of the [(N_5_)_2_BX]^2−^ and [(N_5_)_2_B_2_X_2_]^2−^ (X = H, F, Cl, Br), the borinium anions [(N_5_)_2_BH]^2−^ and [(N_5_)_2_B_2_H_2_]^2−^ are identified as potential synthetic targets of innovative environmentally friendly catalysts.

## 1 Introduction

The chemistry of main-group metallomimetics has been a subject of keen interest for quite some time, leading to the discovery of new reactions and new catalysts.^1^ Metal catalysts are generally expensive and environmentally detrimental, while the main-group metal-free catalysts based on metallomimetics are friendly to the environment and become novel research objects. For the heavy main-group elements, the significant progress of metallomimetics was reviewed by Power in 2010.^2^ Recently the light main-group elements, such as boron with atypical oxidation states, have become attractive candidates for the design of metallomimetics.^3,4^ Classical boron compounds are used as Lewis acids and electrophilic reagents due to their electron deficiency and low electronegativity. Low-valent boron reagents, possessing a single boron(I) active site along with a nonbonding electron pair and two empty p orbitals, behave as Lewis bases with transition-metal-like reactivity.^5^

As early as 1960s the transient borylene [FB:] and its derivative chloroborylene [ClB:] were confirmed to react with alkynes, demonstrating their high reactivity.^6−8^ However, stabilizing borylenes and controlling their reactions in synthetic transformations represent critical challenges that remain to be addressed. The first nucleophilic boryl anion was synthesized by Yamashita and coworkers in 2006.^9^ In 2007, Robinson and coworkers reported the first B=B double bond.^10^ In 2010, Braunschweig systematically summarized the properties of the boron-centered ligands, including the borylene compounds and reported the isolation of the first ever NHC(N-heterocyclic carbene)-stabilized π-nucleophilic boryl anion.^11,12^ Moving a step further, in 2011, Bertrand and coworkers synthesized the first neutral, CAAC-stabilized tricoordinate boron species isoelectronic with amines.^13,14^ However, due to the large steric hindrance around the boron center, most of the electrophiles were hindered. In 2012, Frenking undertook a detailed theoretical study on the structure and bonding of borylene complexes (BH)L_2_ (L = CO, N_2_, PPh_3_, NHC, and CAAC).^15^ In order to further adjust the electronic properties of the boron center, in 2014, Bertrand et al. reported the preparation of unsymmetrically substituted nucleophilic boron derivatives.^16^ In the same year, Kinjo and coworkers installed sterically less demanding oxazol-2-ylidenes substituents at the boron center. Subsequent studies showed that the central boron atom possesses soft Lewis basic nature.^17,18^ Compared with NHCs and CAACs, the nitrogen oxygen heterocycles are more suitable as ligands to stabilize the boron guest. Bertrand, Stephan, et al. reported the synthesis of a stable carbene borylene adduct, which is stabilized by the push pull effect of the amino group and the CAAC ligand.^19^ From 2015 to 2017, Braunschweig et al. reported advancements in borylene chemistry, including the isolation of a stable borylene dicarbonyl, noncluster Lewis adducts, and a CO adduct of a reactive CAAC-bound arylborylene.^20−24^ In 2019, Phukan et al. perform computational studies on a series of bis(carbene) borylene complexes.^25^ Dianions of the type [B_2_X_2_(NR_2_)_2_]^2–^ were isolated by Power et al. in 1992.^26^ Since then a handful of amino-, aryl-, and heteroaryl-substituted dianions^27−29^ have been structurally characterized. In 2023, Braunschweig et al. presented the first example of confirmed borylene-to-diborene dimerization.^30^ In the same year, Braunschweig et al. also discovered the formation of a magnesium complex with an η^5^-diborafulvene dianion.^31^ The B–B single and multiple bonds are also found to be involved in a rich variety of TM-like reactivity.^32−41^ Recently, So et al. reported the synthesis of a diboron compound and its double single-electron-transfer (SET) reactivity in small-molecule activation.^42^

Based on this, we have noticed that a large steric hindrance can affect the interactions between boron reagents and reactants, resulting in stable active sites and reaction inertness. In order to stabilize nucleophilic tricoordinate boron atoms while retaining reactivity toward electrophilic reagents, we choose a small ligand [cyclo-N_5_]^−^, which is an electron-rich system and can be used as a electron donor,^43−45^ similar to the nitrogen–oxygen heterocyclic carbene ligands with p-donor abilities. However, [cyclo-N_5_]^−^ is known to exhibit a relatively low energy barrier (15 kcal/mol) for the loss of N_2_.^46^ Braunschweig et al. proposed the perspective that the B–H bonding pair electrons in boranes show nucleophilicity.^47^ In boron trihalides, the lone pair of electrons on the halogen overlaps with the vacant orbitals on the boron center, resulting in the formation of a  bond, and these compounds are well-known Lewis acids, frequently utilized as catalysts.^48−50^

In the present paper, we will theoretically study the monoboron [(N_5_)_2_BX]^2–^ and diboron [(N_5_)_2_B_2_X_2_]^2–^ (X = H, F, Cl, Br) anionic compounds and discuss their electronic structures and properties, including the π-conjugation, hyperconjugation, nucleophilicity, and the stability. For comparison, we have also carried out computational studies of the anions [BH_3_]^2–^, [BF_3_]^2–^, [B(NH_2_)_3_]^2–^, [(NMe_2_)_2_BH]^2–^, [(NMe_2_)_2_B_2_H_2_]^2–^, [(N_5_)_2_BH], and [(N_5_)_2_B_2_H_2_]·

## 2 Computational Methods

The revDSD-PBEP86 method^51,52^ was employed for the geometry optimizations. The def2-TZVP^53^ basis sets were used for the H, B, N, F, Cl, and Br atoms.

The NBO^54−56^ analysis is performed at the same levels of theory, in order to understand the electronic characters of these compounds. Second-order perturbation interaction energies (*E*^(2)^) for NBOs were examined to measure the effects of conjugation, hyperconjugation, and through-space electron delocalization. Natural atomic charges^57^ of the boron atoms describe the degree of electron transfer from the substituents to the boron center.

Intrinsic reaction coordinate (IRC)^58^ analyses were performed to confirm that the given transition structure connects the reactants and products with the B3LYP method.^59−61^ The 6-31G*^62^ basis sets were used for the H, B, N, F, and Cl atoms, and the Lanl08(d)^63^ basis set with the ECP core for the Br atom. The transition state energy barrier calculation corresponding to the IRC was performed using the revDSD-PBEP86 method^51,52^ and the def2-TZVP^53^ basis sets. All of these computations were carried out with the Gaussian 16 program.^64^

Based on the revDSD-PBEP86/def2-TZVP results, the wave function analyses were performed with the Multiwfn code [version 3.8 (dev)],^65,66^ and the isosurfaces of various real space functions were rendered by the Visual Molecular Dynamics (VMD) software.^67^ The color-filled maps of various real space functions were plotted directly using the Multiwfn code.

## 3 Results and Discussion

### 3.1 Geometrical Structures and Stability

**Figure 1:**
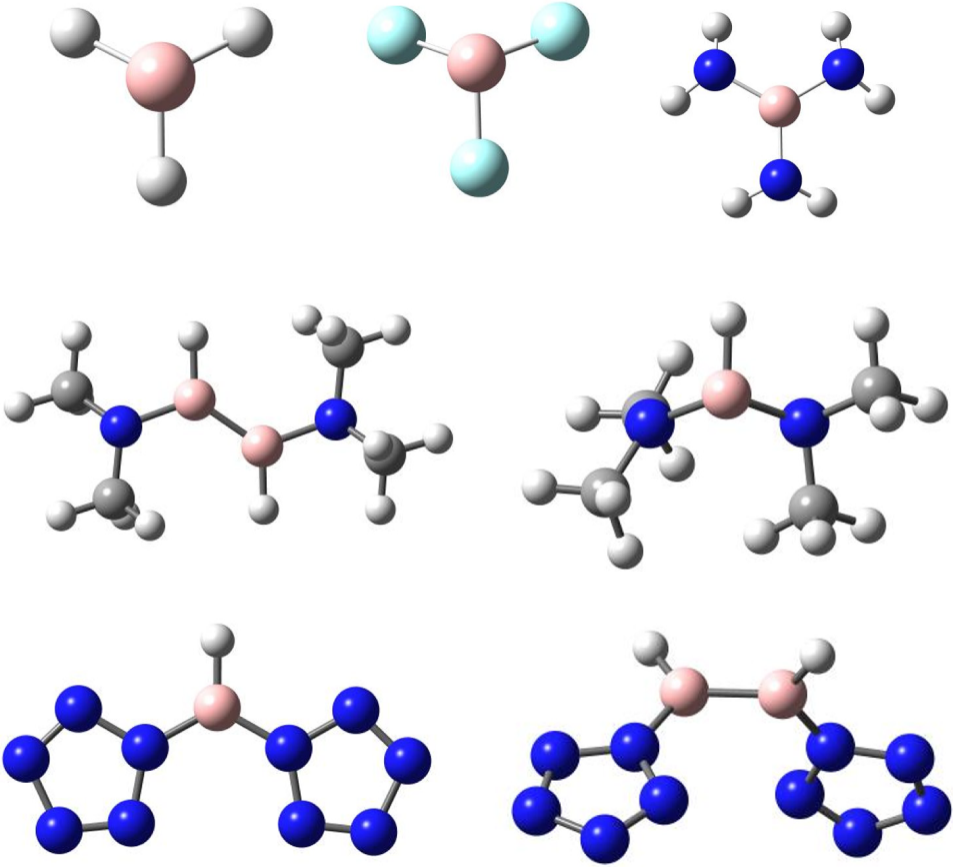
Optimized structures for compounds [BH_3_]^2–^, [BF_3_]^2–^, [B(NH_2_)_3_]^2–^, [(NMe_2_)_2_BH]^2–^, [(NMe_2_)_2_B_2_H_2_]^2–^, [(N_5_)_2_BH], and [(N_5_)_2_B_2_H_2_]·.

The optimized structures of [BH_3_]^2–^, [BF_3_]^2–^, [B(NH_2_)_3_]^2–^, [BH(NMe_2_)_2_]^2–^, [B_2_H_2_(NMe_2_)_2_]^2–^, [(N_5_)_2_BH], and [(N_5_)_2_B_2_H_2_] are shown in Figure 1. The B–X distances and NBO results for these compounds are reported in Tables 1 and 2.

**Table 1:** B–X Distances, Wiberg Bond Indices (WBI), Natural Atomic Charges on B and X Atoms (*q*_*B*_ and *q*_*x*_), and Transition State Barriers for Ligand Dissociation (*E*_TS_) of [BH_3_]^2–^, [BF_3_]^2–^, and [B(NH_2_)_3_]^2–^

System		B–X length (Å)	B–X WBI	NPA		*E*_TS_ (kcal/mol)
				*q*_*B*_	*q*_*x*_	
[BH_3_]^2–^	*C*_3*v*_	1.262	0.99	–1.61	–0.13	24.7
[BF_3_]^2–^	*C*_3*v*_	1.489	0.53	+0.11	–0.70	6.7
[B(NH_2_)]^2–^	*C*_3*v*_	1.463	0.86	+0.35	–1.39	18.2

**Table 2:** B–B and B–X Distances, Wiberg Bond Indices (WBI), and Natural Atomic Charges on B and X Atoms (*q*_*B*_ and *q*_*x*_), Transition State Barriers for N_5_ Ring Breakups (*E*_TS_) of [(N_5_)_2_BH] and [(N_5_)_2_B_2_H_2_] and Transition State Barriers for Ligand Dissociation (*E*_TS_) of [BH(NMe_2_)_2_]^2–^ and [B_2_H_2_(NMe_2_)_2_]^2–^

System		B–B length (Å)	B–X length (Å)	NBBN dihedral angles	XBBX dihedral angles	B–B WBI	NPA		*E*_TS_ (kcal/mol)
							*q*_*B*_	*q*_*x*_	
[(NMe_2_)_2_BH]^2–^	*C*_1_		1.247				–0.29	–0.19	9.0
[(N_5_)_2_BH]	*C*_2*v*_		1.177				+0.88	–0.03	11.0
[(NMe_2_)_2_B_2_H_2_]^2–^	*C*_*i*_	1.624	1.241	180	180	1.82	–0.27	–0.15	40.5
[(N_5_)_2_B_2_H_2_]	*C*_2_	1.687	1.187	90	87	0.99	+0.48	–0.04	11.6

The symmetries of [BH_3_]^2–^, [BF_3_]^2–^, and [B(NH_2_)_3_]^2–^ are all C_3v_, resembling the isoelectronic trigonal pyramidal geometry of NH_3_. This is due to the presence of two negative charges, which cause the structure to deviate from a planar arrangement to a trigonal pyramidal one. According to the B–X WBI (Table 1), it is evident that both X = F and NH_2_ have values less than 1, and the bonds are weaker compared to X = H. Furthermore, the relative magnitudes of the B–X WBI values are as follows: F < NH_2_ < H. This trend corresponds to the transition state energies for the dissociation of ligands (H, F, NH_2_) from boron, where F < NH_2_ < H (6.7 < 18.2 < 24.7). Thus, it can be concluded that [BH_3_]^2–^ is the most stable of the three. However, the transition state energy of [BH_3_]^2–^ is 24.7 kcal/mol, which is lower than that of [(N_5_)_2_BH]^2–^, namely, 26.2 kcal/mol.

The species [(NMe_2_)_2_BH]^2–^ is predicted to be a local minimum with C_1_ symmetry on its potential energy surface. The two [NMe_2_]^−^ groups are approximately perpendicular, and the natural population analysis (NPA) charge on the boron atom is −0.29, which is smaller than the minimum value of 0.20 for the N_5_^–^ ligand (X = H). However, the transition state energy for the departure of [NMe_2_]^−^ from boron is only 9 kcal/mol, indicating that [(NMe_2_)_2_BH]^2–^ is highly unstable.

On the other hand, [(NMe_2_)_2_B_2_H_2_]^2–^ exhibits *C_i_* symmetry with a symmetry center. The two [NMe_2_]^−^ groups are nearly parallel, with both the NBBN and XBBX dihedral angles being 180 deg. The NPA charge on the boron atom is −0.29, which is smaller than the minimum value of −0.18 for the N_5_^–^ ligand (X = H). The B–B Wiberg bond index (WBI) is 1.82, which is larger than the maximum value of 1.69 for the N_5_^–^ ligand (X = Br), suggesting that the B–B bond strength in [(NMe_2_)_2_B_2_H_2_]^2–^ is greater. The transition state energy for the departure of [NMe_2_]^−^ from boron is as high as 40 kcal/mol, which is attributed to the steric hindrance provided by the [NMe_2_]^−^ groups that stabilize the boron center.

The neutral [(N_5_)_2_BH] and [(N_5_)_2_BH]^2–^ molecules both adopt *C*2_*v*_ symmetry. Compared to [(N_5_)_2_BH]^2–^, which carries two negative charges, the natural population analysis (NPA) charge on the boron atom in [(N_5_)_2_BH] increases from +0.20 to +0.88. The transition state barrier for the N_5_^–^ ring breaking into N_2_ and N_3_ is 11.0 kcal/mol, indicating that neutral [(N_5_)_2_BH] is less stable than [(N_5_)_2_BH]^2–^.

The symmetry of [(N_5_)_2_B_2_H_2_] is *C*_2_, with reduced symmetry compared to the *C*2_*h*_ symmetry of [(N_5_)_2_B_2_H_2_]^2–^. The NBBN and XBBX dihedral angles in [(N_5_)_2_B_2_H_2_] are approximately 90 deg, while in [(N_5_)_2_B_2_H_2_]^2–^, both dihedral angles are 180 deg, transitioning from parallel to perpendicular. Compared to [(N_5_)_2_B_2_H_2_]^2–^, the B–B bond length in [(N_5_)_2_B_2_H_2_] increases from 1.589 to 1.687 Å, while the corresponding Wiberg bond index (WBI) for the B–B bond decreases from 1.66 to 0.99. The B–B bond in [(N_5_)_2_B_2_H_2_] is single. Similar to that in monoboron compounds, the NPA charge on the boron atom in [(N_5_)_2_B_2_H_2_] increases from −0.18 to +0.48, The transition state barrier for the N_5_^–^ ring breaking into N_2_ and N_3_ is 11.6 kcal/mol, which is also smaller than that for [(N_5_)_2_B_2_H_2_]^2–^, indicating that [(N_5_)_2_B_2_H_2_]^2–^ is more stable than [(N_5_)_2_B_2_H_2_].

**Figure 2:**
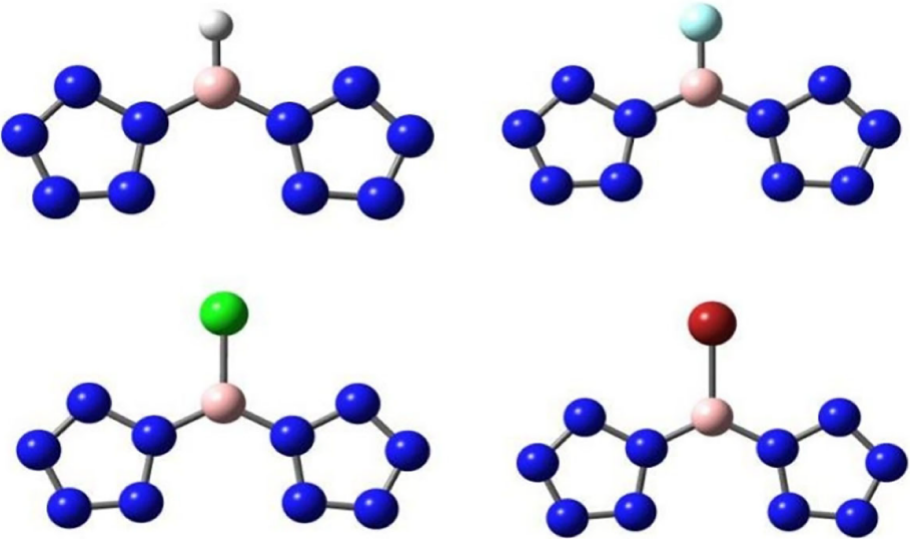
Optimized structures for compounds [(N_5_)_2_BX]^2–^ (X = H, F, Cl, and Br).

The optimized structures of [(N_5_)_2_BX]^2–^ (X = H, F, Cl, and Br) are shown in Figure 2. The B–X distances and NBO results for these compounds are reported in Table 3.

**Table 3:** B–B and B–X Distances, Wiberg Bond Indices (WBI), Natural Atomic Charges on B and X Atoms (*q*_*B*_ and *q*_*x*_)

System		B–B length (Å)	B–X length (Å)	NBBN dihedral angles	XBBX dihedral angles	B–B WBI	NPA	
							*q*_*B*_	*q*_*x*_
[(N_5_)_2_BH]^2–^	*C*_2*v*_		1.195				+0.20	–0.09
[(N_5_)_2_BF]^2–^	*C*_2*v*_		1.382				+0.83	–0.54
[(N_5_)_2_BCl]^2–^	*C*_2*v*_		1.821				+0.42	–0.30
[(N_5_)_2_BBr]^2–^	*C*_2*v*_		1.983				+0.36	–0.28
[(N_5_)_2_B_2_H_2_]^2–^	*C*_2*h*_	1.589	1.209	180	180	1.66	–0.18	–0.08
[(N_5_)_2_B_2_F_2_]^2–^	*C*_2*h*_	1.603	1.392	180	180	1.48	+0.36	–0.56
[(N_5_)_2_B_2_Cl_2_]^2–^	*C*_2_	1.580	1.849	179	174	1.60	+0.03	–0.31
[(N_5_)_2_B_2_Br_2_]^2–^	*C*_2_	1.563	2.024	179	171	1.69	–0.04	–0.29

**Figure 3:**
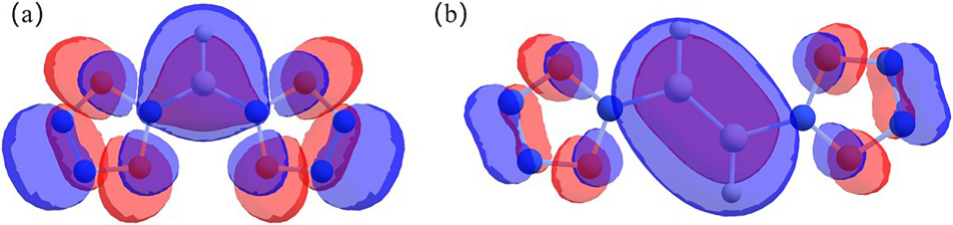
HOMO values of (a) [(N_5_)_2_BH]^2–^ and (b) [(N_5_)_2_B_2_H_2_]^2–^.

All of the [(N_5_)_2_BX]^2–^ (X = H, F, Cl, and Br) compounds are predicted to be local minima with *C*2_*v*_ symmetry on their potential energy surface. As shown in Figure 2, these compounds are composed of two N_5_^–^ rings connected by the BX fragment. In the N_5_^–^ rings, each N atom is sp^2^ hybridized with its *p*_*z*_ orbital perpendicular to the ring plane to take part in the delocalized  bond. All the N_5_^–^ rings in the [(N_5_)_2_BX]^2–^ compounds display geometries similar to the isolated N_5_^–^ anion. The central B atom is also involved in sp^2^ hybridization. Two empty in-plane sp^2^ orbitals accept lone pairs from the two [cyclo-N_5_]^−^ rings to form two dative bonds. One singly occupied sp^2^ orbital forms a normal covalent bond with the X atom. The other two valence electrons of boron remain in the *p*_*z*_ orbital as a lone pair perpendicular to the molecular plane. With 8 electrons in the valence layer of boron satisfying the octet rule, the [(N_5_)_2_BX]^2–^ compounds may be more stable than the classical boron Lewis acid. In addition, the HOMO of [(N_5_)_2_BH]^2–^ shows that the conjugation of the *p*_*z*_ electrons is delocalized over the entire molecule (Figure 3a), and the 14 conjugated π electrons just meet the Hückel 4*n* + 2 rule to stabilize this molecule. The NBO analyses show that the NPA atomic charges on the X atom in the [(N_5_)_2_BX]^2–^ compounds are −0.09, −0.28, −0.30, and −0.54 (Table 3) for X = H, Br, Cl, and F, respectively, consistent with the electronegativities of the X atoms, increasing from H to Br, Cl, and F. Since the atomic charges on the B atom decrease accordingly, the natural charges for the −BX group are small, i.e., from 0.08 (−BBr) to 0.30 (−BF). Thus, the charges on all of the N_5_^–^ rings in different [(N_5_)_2_BX]^2–^ compounds remain close to −1.0, i.e., from −1.04 (X = Br) to −1.15 (X = F).

**Figure 4:**
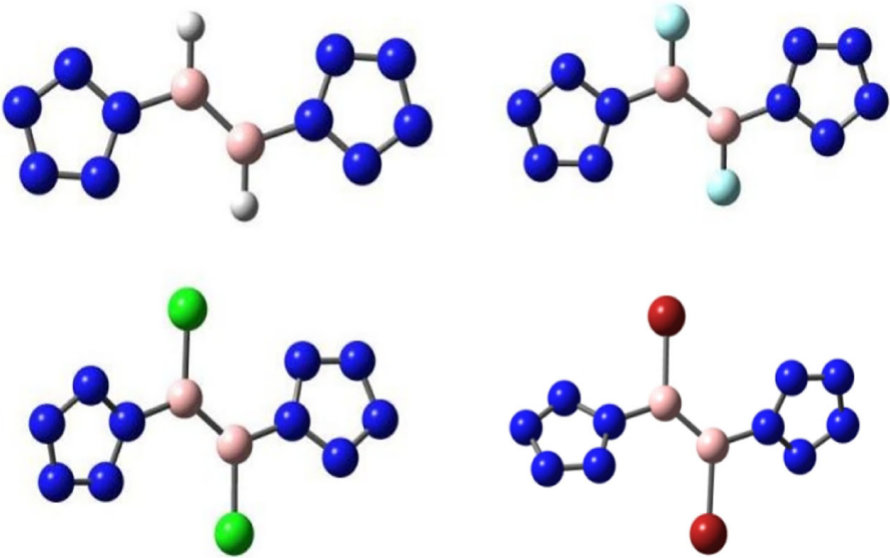
Optimized structures for the compounds [(N_5_)_2_B_2_X_2_]^2–^ (X = H, F, Cl, and Br).

The optimized structures of [(N_5_)_2_B_2_X_2_]^2–^ (X = H, F, Cl, Br) are shown in Figure 4, and some bond distances and NBO results for these compounds are also reported in Table 3. Among these, [(N_5_)_2_B_2_H_2_]^2–^ and [(N_5_)_2_B_2_F_2_]^2–^ are planar with *C*2_*h*_ symmetry, while [(N_5_)_2_B_2_Cl_2_]^2–^ and [(N_5_)_2_B_2_Br_2_]^2–^ are slightly out-of-plane with *C*_2_ symmetry. The XBBX dihedral angles are 174° for [(N_5_)_2_B_2_Cl_2_]^2–^ and 171° for [(N_5_)_2_B_2_Br_2_]^2–^. The electronic structures for the [(N_5_)_2_B_2_X_2_]^2–^ compounds are somewhat different from those for the monoboron [(N_5_)_2_B_2_X_2_]^2–^ compounds. The two B atoms form two dative bonds with two N_5_^–^ rings, respectively. Also, there are two B–X σ bonds and one σ B–B bond. The singly occupied electron in the *p*_*z*_ orbital of each boron atom could form delocalized conjugated π bond with the π systems of the two N_5_^–^ rings. Actually, Figure 3b shows that the HOMO of [(N_5_)_2_B_2_H_2_]^2–^ is a π orbital delocalized over the entire molecule containing 14 π electrons, satisfying the Hückel rule and stabilizing these compounds. The NPA atomic charge on each B atom in [(N_5_)_2_B_2_X_2_]^2–^ is more negative (or less positive) than those in the monoboron [(N_5_)_2_BX]^2–^ compounds, i.e., −0.18, −0.04, 0.03, and 0.36 (Table 3) for X = H, Br, Cl, and F, respectively. This is because there is one less N_5_^–^ ring to draw electrons from the B atom. This makes the [(N_5_)_2_B_2_X_2_]^2–^ compounds more favorite candidates as low-valent boron reagents. Similar to [(N_5_)_2_BH]^2–^, the −BX group charges in [(N_5_)_2_B_2_X_2_]^2–^ are still trivial (but more negative), from −0.20 (−BF) to −0.33 (−BBr). The natural charges on the N_5_^–^ rings are not far from −1.0, i.e., from −0.67 (X = Br) to −0.80 (X = F).

Table 3 shows that the B–B distances in [(N_5_)_2_B_2_X_2_]^2–^ (X = H, F, Cl, and Br) are 1.56–1.60 Å, namely, shorter than that of the normal B–B single bond (1.64 Å). Accordingly the WBI B–B values are predicted to be about 1.5, suggesting that the B–B π bond order in this conjugation system is less than one. The B–X (X = H, F, Cl, Br) bond distances in [(N_5_)_2_B_2_X_2_]^2–^ are similar to those in [(N_5_)_2_BX]^2–^. For instance, the B–H bond in [(N_5_)_2_B_2_H_2_]^2–^ is 1.209 Å, while the B–H bond in [(N_5_)_2_BH]^2–^ is 1.195 Å, which is only 0.01 Å shorter. The NPA atomic charge on each B atom in [(N_5_)_2_B_2_X_2_]^2–^ ranges from −0.18 to 0.36. These results are consistently more negative than that in [(N_5_)_2_BX]^2–^, due to one less N_5_^–^ ring to draw electrons from it. This makes the [(N_5_)_2_B_2_X_2_]^2–^ compounds more favorable candidates as low-valent boron reagents.

**Figure 5:**
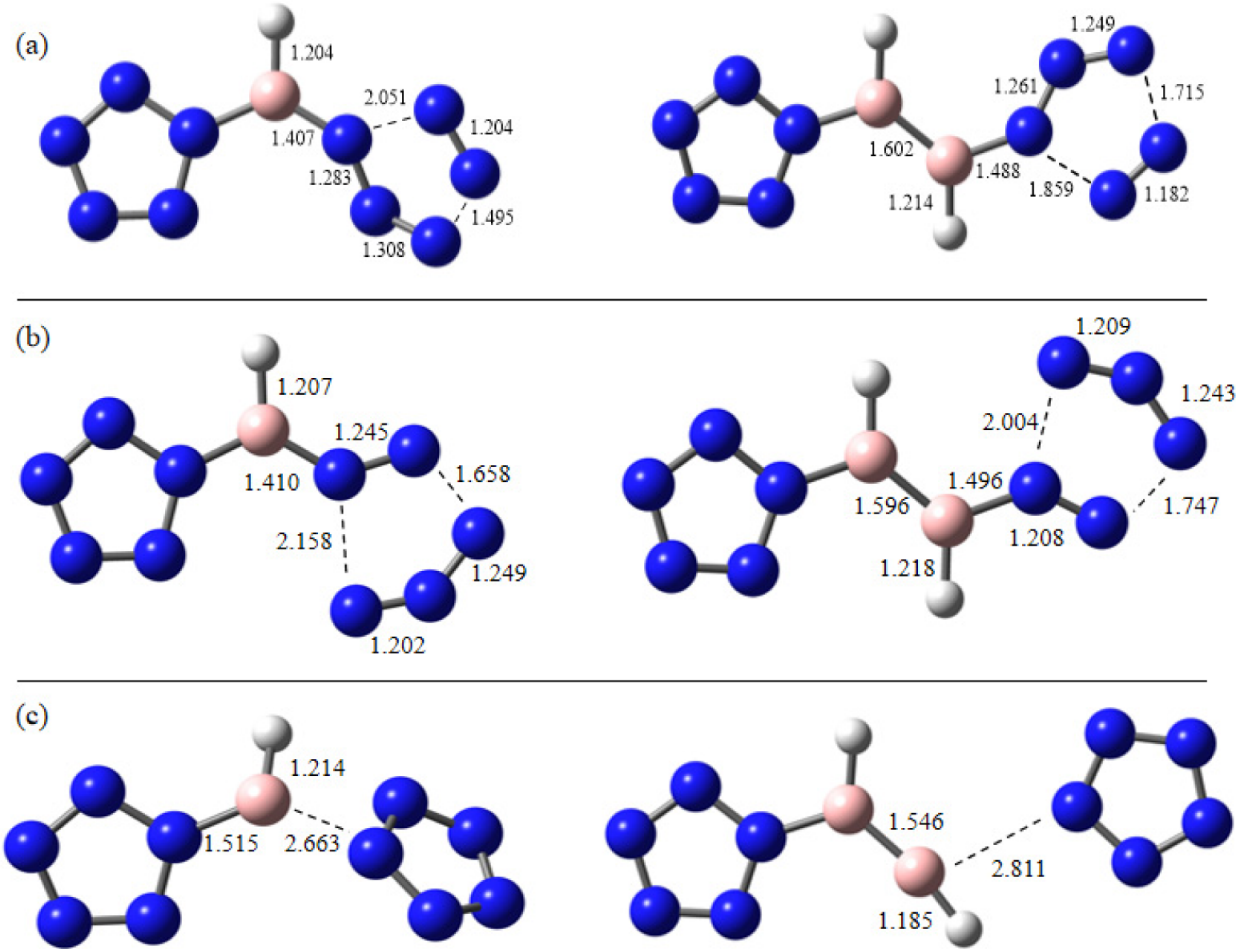
Selected transition states for [(N_5_)_2_BH]^2–^ and [(N_5_)_2_B_2_H_2_]^2–^.

The kinetic stabilities of the title compounds can be analyzed by identifying three transition states: TSa, TSb, and TSc (Table 4), which correspond to the transition states shown in Figure 5a, b, and c, respectively. TSa represents the transition state for the removal of N_2_ from the N_5_^–^ ring, TSb corresponds to the transition state for the removal of N_3_, and TSc pertains to the transition state for the dissociation of the B–N_5_^–^ bonds. The TS barriers (shown in Table 4) for the [(N_5_)_2_BX]^2–^ and [(N_5_)_2_B_2_X_2_]^2–^ (X = H, F, Cl, and Br) compounds are all above 20 kcal/mol, except for [(N_5_)_2_BF]^2–^, for which the *E*_TSc_ is 19.7 kcal/mol. The *E*_TSa_ values for [(N_5_)_2_BX]^2–^ and [(N_5_)_2_B_2_X_2_]^2–^ (X = H, F, Cl, and Br) are all higher than the energy barrier (15 kcal/mol) for the loss of N_2_ from the N_5_^–^ ring.^46^ By selecting the minimum value from the three transition states for each compound, the following ranking is obtained: for [(N_5_)_2_BX]^2–^ (X = H, F, Cl, and Br), [(N_5_)_2_BH]^2–^ has the highest barrier at 26.2 kcal/mol, while for [(N_5_)_2_B_2_X_2_]^2–^ (X = H, F, Cl, and Br), [(N_5_)_2_B_2_F_2_]^2–^ has the highest barrier at 27.5 kcal/mol, with [(N_5_)_2_B_2_H_2_]^2–^ is the second highest, differing by 0.7 kcal/mol. For the three transition states of the same compound, the lowest energy is typically observed for TSa, indicating that the N_5_^–^ ring losing N_2_ is the most kinetically favorable pathway.

**Table 4:** Transition State Barriers for N_5_ Ring Breakups (*E*_TSa_ and *E*_TSb_) and the Dissociations of the B–N_5_ Bonds (*E*_TSc_) of the Title Compounds

System	*E*_TSa_ (kcal/mol)	*E*_TSb_ (kcal/mol)	*E*_TSc_ (kcal/mol)
[(N_5_)_2_BH]^2–^	26.2	26.5	34.6
[(N_5_)_2_BF]^2–^	23.2	24.8	19.7
[(N_5_)_2_BCl]^2–^	23.3	24.1	26.4
[(N_5_)_2_BBr]^2–^	23.3	23.9	27.1
[(N_5_)_2_B_2_H_2_]^2–^	28.9	31.8	26.8
[(N_5_)_2_B_2_F_2_]^2–^	27.5	28.9	27.9
[(N_5_)_2_B_2_Cl_2_]^2–^	23.9	30.8	33.1
[(N_5_)_2_B_2_Br_2_]^2–^	21.6	30.6	36.4

### 3.2 Electron Delocalization

To explain the π electron distribution differences between [(N_5_)_2_BH]^2–^ and [(N_5_)_2_B_2_H_2_]^2–^, we focus on their global π electron delocalization characteristics in this section. The localized orbital locator (LOL) is a popular real space function used to exhibit the degree of electron delocalization for chemical systems in three-dimensional space.^68,69^ In this section, the LOL function is employed to illustrate graphically the electron delocalization of the representative title compounds.

**Figure 6:**
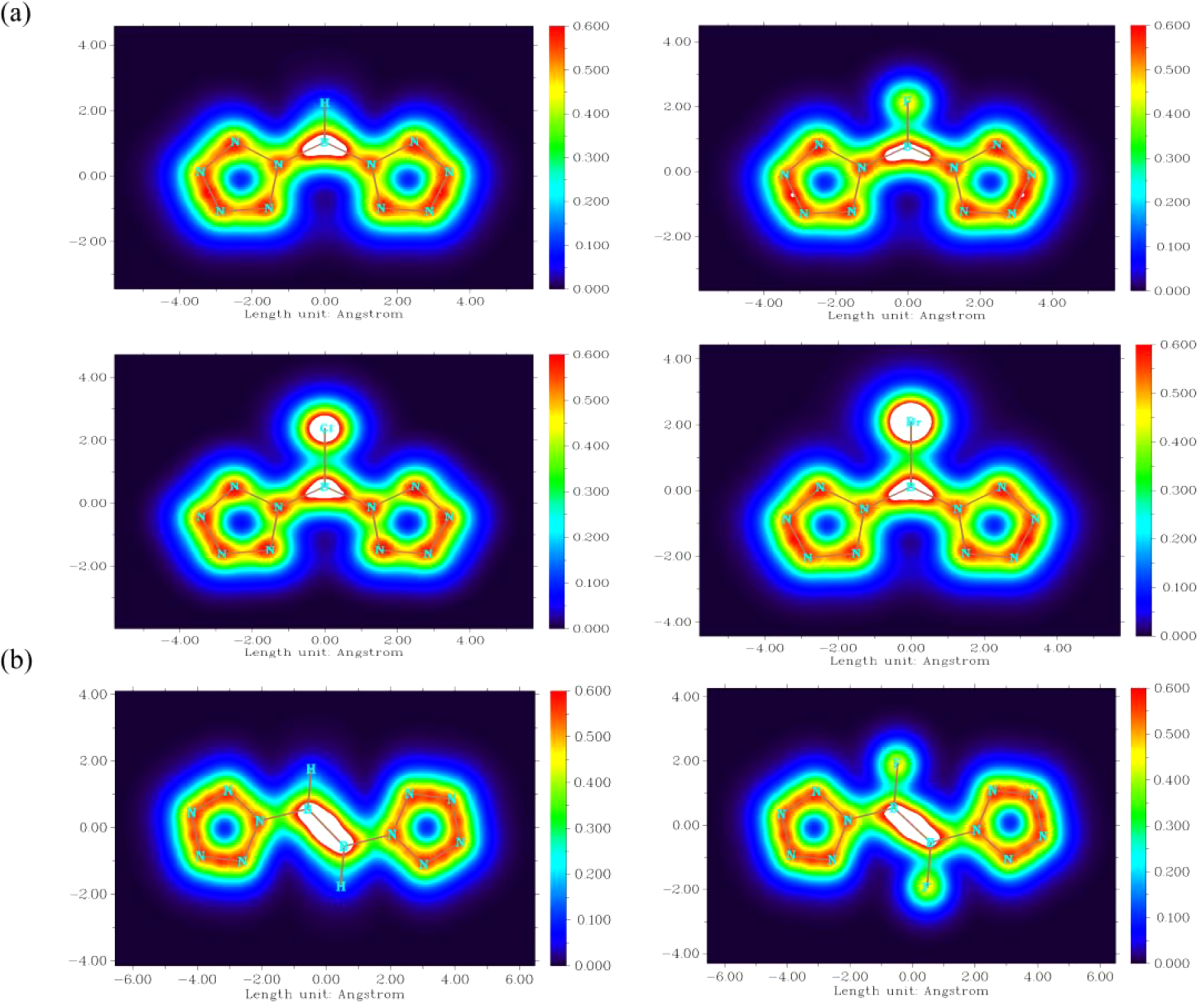
(a) Color-filled map of LOL-π at 1.2 bohr above [(N_5_)_2_BX]^2–^ (X = H, F, Cl, and Br); (b) color-filled map of LOL-π at 1.2 bohr above [(N_5_)_2_B_2_H_2_]^2–^and [(N_5_)_2_B_2_F_2_]^2–^. The white color indicates values that exceed the upper limit of the color scale.

The color-filled maps of LOL-π at 1.2 bohr above the planes of the [(N_5_)_2_BX]^2–^ (X = H, F, Cl, Br) and [(N_5_)_2_B_2_X_2_]^2–^ (X = H, F) molecules are plotted in Figure 6, representing the π electron delocalization. These contour maps show that the B atom and two N_5_^–^ rings form delocalized π bonds, indicating a strong conjugation effect. Figure 6a shows that for [(N_5_)_2_BH]^2–^, there is a stable conjugation system with 14 π electrons. However, for the [(N_5_)_2_BX]^2–^ (X = F, Cl, Br) molecules, both the boron center and halogen regions have high π electron densities, indicating a weaker conjugation effect between the π electrons on the halogen atoms and the conjugation system over the B atom and two N_5_^–^ rings, which have favorable 14 π electrons.

**Figure 7:**
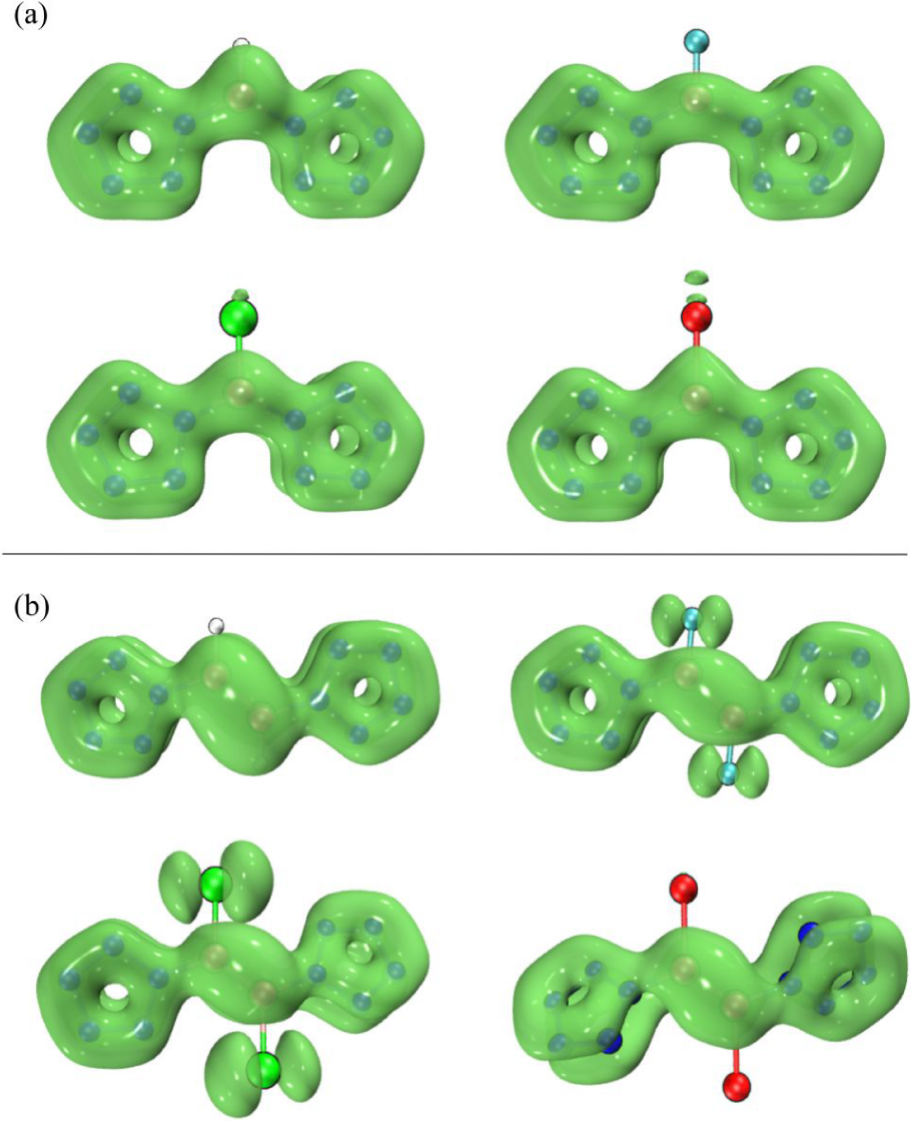
LOL-π isosurfaces (isovalue = 0.2) for (a) [(N_5_)_2_BX]^2–^ and (b) [(N_5_)_2_B_2_X_2_]^2–^ (X = H, F, Cl, and Br).

Figure 7 describes the LOL-π isosurfaces (isovalue of 0.2) of [(N_5_)_2_BX]^2–^ and [(N_5_)_2_B_2_X_2_]^2–^ (X = H, F, Cl, and Br). These show that the conjugative interaction plays a very important role for stabilizing boron. In [(N_5_)_2_BX]^2–^ and [(N_5_)_2_B_2_X_2_]^2–^ (X = F, Cl, and Br), there is no conjugation between the X atom and the conjugated system of B and N_5_^–^. The lone pairs on the X atom may fill the antibonding π orbitals due to the high electronegativity and strong electron-attracting force of the halogen atoms (F, Cl, and Br). This weakens the delocalization of π electrons in [(N_5_)_2_BX]^2–^ and [(N_5_)_2_B_2_X_2_]^2–^ (X = F, Cl, and Br). In comparison, [(N_5_)_2_BH]^2–^ and [(N_5_)_2_B_2_H_2_]^2–^ are relatively stable without antibonding π orbitals in the 14-electron conjugation system. The hydrogen atoms have lower electronegativity and do not excessively attract electrons, leading to 14-π-electron delocalization all over the [(N_5_)_2_BH]^2–^ and [(N_5_)_2_B_2_H_2_]^2–^ molecules, indicating that they are most likely to be synthesized.

**Figure 8:**
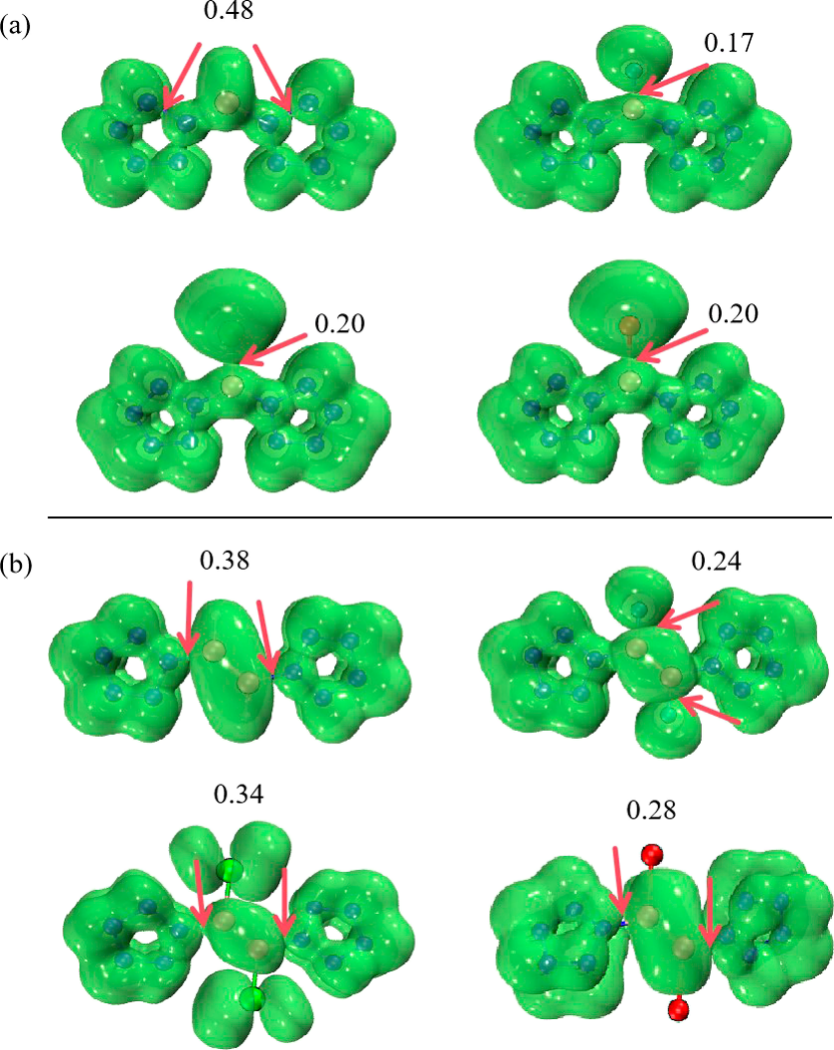
Isosurface maps of ELF-π for (a) [(N_5_)_2_BX]^2–^ and (b) [(N_5_)_2_B_2_X_2_]^2–^ (X = H, F, Cl, and Br). The values taken by the isovalue are the basis values labeled on the graph.

The values of bifurcation points on the electron localization function (ELF) isosurface are valuable in quantifying the degree of π conjugation.^70,71^ The bifurcation points of ELF-π correspond to the positions at which a connected ELF-π isosurface begins to split into two as the isovalue increases. The isosurface maps of ELF-π for [(N_5_)_2_BX]^2–^ and [(N_5_)_2_B_2_X_2_]^2–^ (X = H, F, Cl, and Br) are shown in Figure 8. The ELF-π maps show that for [(N_5_)_2_BX]^2–^ (X = H, F, Cl, and Br), the bifurcation points for X = F, Cl, and Br are located between X and B, whereas for [(N_5_)_2_BH]^2–^, the bifurcation points are between the nitrogen atoms connected to boron and their adjacent nitrogen atoms. This correlates with the observations in Figure 6a, where the region between the two nitrogen atoms at the breaking point in [(N_5_)_2_BH]^2–^ is yellow, while the remaining two nitrogen atoms on N_5_^–^ are red. For X = F, Cl, and Br, the region between X and B is colored green. The values of ELF-π for [(N_5_)_2_BX]^2–^ (X = F, Cl, Br) are 0.17, 0.20, and 0.20, respectively. Since the values of ELF-π for antiaromatic compounds fall in the range of 0.11–0.35,^72^ these three compounds exhibit antiaromatic character. In contrast, the value of ELF-π for [(N_5_)_2_BX]^2–^ is 0.48, which is greater than 0.35, indicating the absence of antiaromaticity. This result is consistent with the observations from the LOL-π isosurfaces. For [(N_5_)_2_B_2_X_2_]^2–^ (X = F, Cl, Br), the bifurcation points for X = F, Cl, and Br are located between X and N_5_^–^. For [(N_5_)_2_B_2_F_2_]^2–^, the bifurcation points occur between F and B, corresponding to the color distribution seen in the LOL-π isosurfaces. Similarly, the values of ELF-π for [(N_5_)_2_B_2_X_2_]^2–^ (X = F, Cl, Br) are 0.24, 0.34, and 0.28, respectively, indicating that these three compounds also exhibit antiaromaticity. In contrast, the value of ELF-π for [(N_5_)_2_B_2_H_2_]^2–^ is 0.48, greater than 0.35, suggesting that it does not exhibit antiaromaticity, consistent with the LOL-π isosurface observations. The analysis of the ELF function provides quantitative evidence for the above LOL analysis.

### 3.3 NBO Analysis

NBO second order perturbative energy *E*^(2)^ analyses reveal especially large conjugative and hyperconjugative effects for the title compounds. Table 5 shows the comparison of the interaction σ(B6H12) → σ*(N4N5) for the [(N_5_)_2_BX]^2–^ (X = H, F, Cl, Br) compounds. The energy order is F < Cl < Br < H, which has a negative correlation with their electronegativities. Similarly, the ordering of the σ(B11H14) → σ*(N8N9) interactions for [(N_5_)_2_B_2_X_2_]^2–^ (X = H, F, Cl, and Br) is also F < Br ∼ Cl < H. Thus, among the title compounds, the X = H species is particularly favorable for stabilizing boron, and hereafter we will focus on the (hyper)conjugation of [(N_5_)_2_BH]^2–^ and [(N_5_)_2_B_2_H_2_]^2–^.

**Table 5:** Comparison of the NBO Second-Order Perturbation Theory Energies *E*^(2)^ (in kcal/mol) for σ(B6X12) → σ*(N4N5) in [(N_5_)_2_BX]^2–^ and σ(B11X14) → σ*(N8N9) in [(N_5_)_2_B_2_X_2_]^2–^ (X = H, F, Cl, and Br)^a^

System	σ(B6H12) → σ*(N4N5)	System	σ(B11H14) → σ*(N8N9)
[(N_5_)_2_BH]^2–^	–6.9	[(N_5_)_2_B_2_H_2_]^2–^	–7.5
[(N_5_)_2_BF]^2–^	–2.0	[(N_5_)_2_B_2_F_2_]^2–^	–2.1
[(N_5_)_2_BCl]^2–^	–3.7	[(N_5_)_2_B_2_Cl_2_]^2–^	–3.4
[(N_5_)_2_BBr]^2–^	–4.6	[(N_5_)_2_B_2_Br_2_]^2–^	–3.2

a Refer to Figures 7 and 8 for atom numberings.

**Figure 9:**
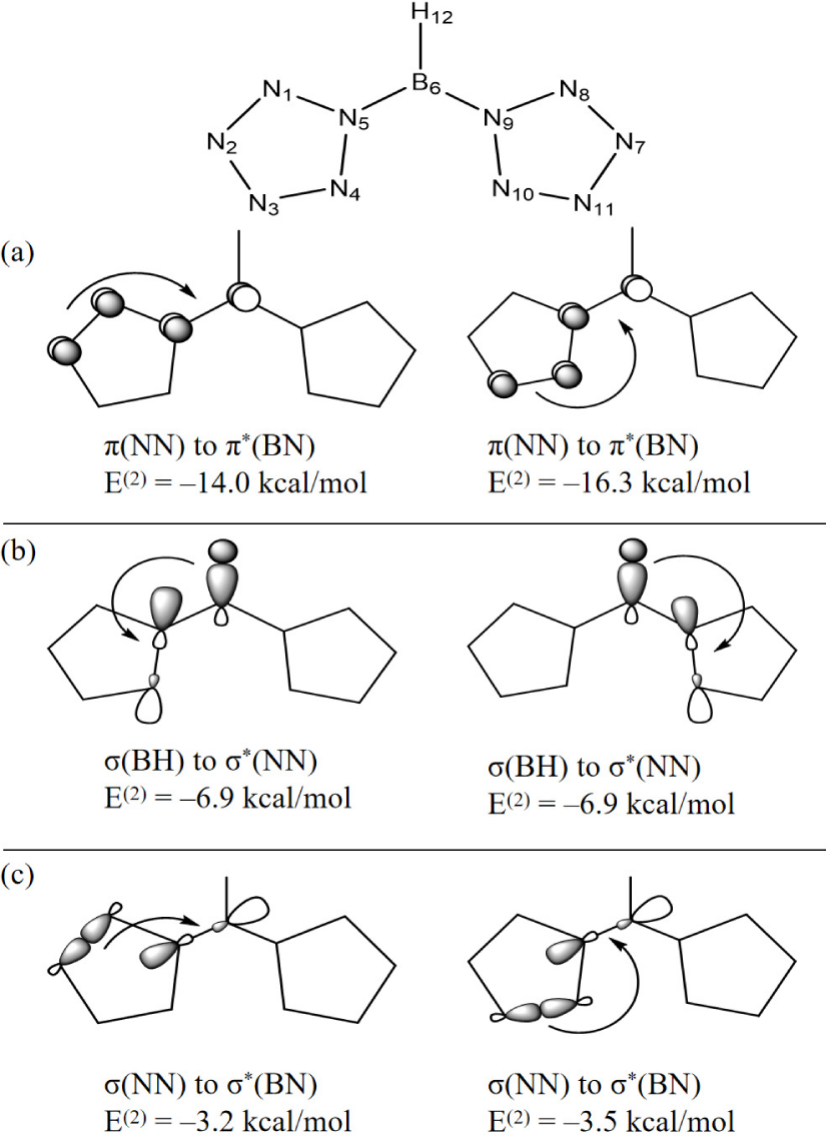
Schematic illustration of (a) conjugative and (b,c) hyperconjugative interactions stabilizing the B center of *C*_2*v*_ [(N_5_)_2_BH]^2–^, based on E2PERT analyses.

In [(N_5_)_2_BH]^2–^, since N1N2 and N3N4 are within the same ring and their interactions are of similar magnitude, the interactions of N1N2 are not depicted in Figure 9. Table 6 and Figure 9 show π-conjugative interactions *E*^(2)^ = −14.0 kcal/mol for π(N3N4) → π*(B5N6), and *E*^(2)^ = −16.3 kcal/mol for π(N3N4) → π*(B5N6). There are two equivalent sets of hyperconjugative interactions between the σ(BH) and σ*(NN) bonds, e.g., *E*^(2)^ = −6.9 kcal/mol for σ(B6H12) → σ*(N4N5) and for σ(B6H12) → σ*(N9N10). Additional stabilizing effects come from hyperconjugative interactions between σ(NN) and σ*(BN), e.g., *E*^(2)^ = −3.2 kcal/mol for σ(N1N2) → σ*(B6N5) and *E*^(2)^ = −3.5 kcal/mol for σ(N3N4) → σ*(B6N5).

**Table 6:** (Hyper)Conjugations and the NBO Second-Order Perturbation Theory Energies *E*^(2)^ (in kcal/mol) for [(N_5_)_2_BH]^2–^ and [(N_5_)_2_B_2_H_2_]^2–^a

[(N_5_)_2_BH]^2–^		[(N_5_)_2_B_2_H_2_]^2–^	
(hyper)conjugation	*E*^(2)^	(hyper)conjugation	*E*^(2)^
π(N1N2) → π*(N5B6)	–14.0	σ(N6N7) → σ*(N8B11)	–4.6
π(N3N4) → π*(N5B6)	–16.3	σ(N9N10) → σ*(N8B11)	–4.5
σ(B6H12) → σ*(N4N5)	–6.9	σ(B11B12) → σ*(N7N8)	–5.8
σ(B6H12) → σ*(N9N10)	–6.9	σ(B11H14) → σ*(N8N9)	–7.5
σ(N1N2) → σ*(N5B6)	–3.2	LP(N8) → σ*(B11B12)	–11.1
σ(N3N4) → σ*(N5B6)	–3.5		

a Refer to Figures 7 and 8 for atom numberings.

**Figure 10:**
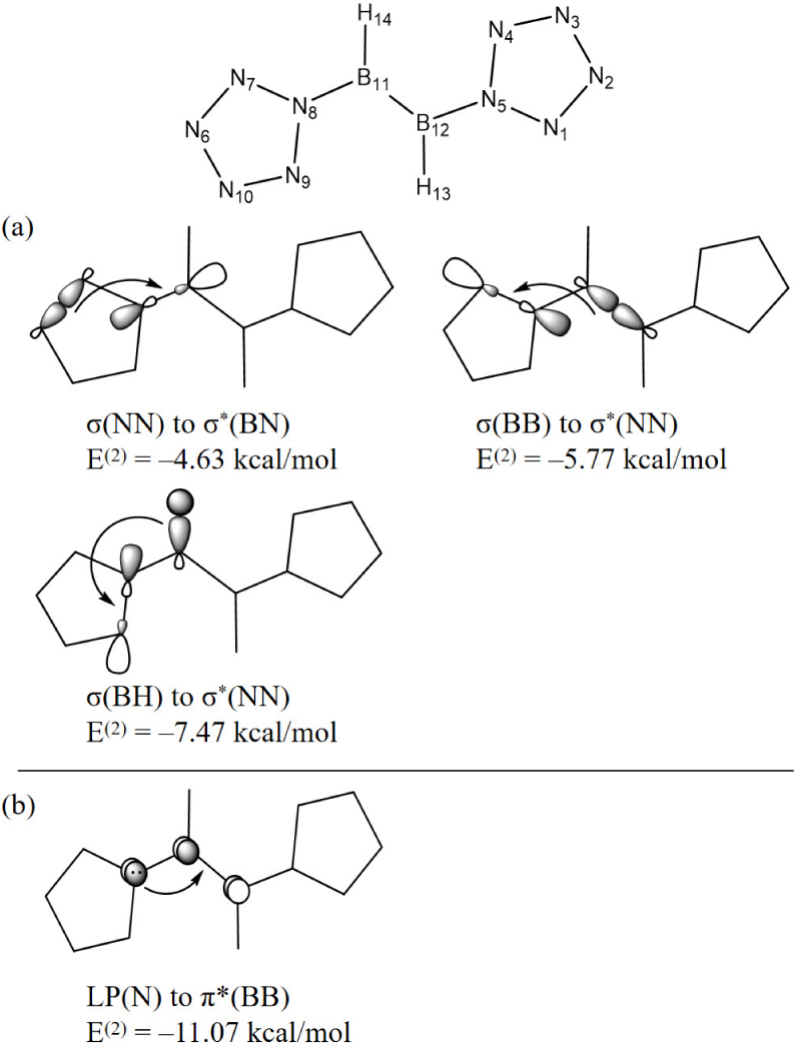
Schematic illustration of (a) hyperconjugative and (b) delocalized π-bonding in *C*_2*h*_ [(N_5_)_2_B_2_X_2_]^2–^ (X = H).

NBO second order perturbation energy *E*^(2)^ analyses for the substituted [(N_5_)_2_B_2_H_2_]^2–^ are shown in Table 6 and Figure 10. In [(N_5_)_2_B_2_H_2_]^2–^, the B center is stabilized by hyperconjugative σ(NN) → σ*(BN). That is, *E*^(2)^ = −4.6 kcal/mol for σ(N6N7) → σ*(B11N8), and *E*^(2)^ = −4.5 kcal/mol for σ(N9N10) → σ*(B11N8), as well as two sets in other ring by symmetry. Also by hyperconjugative σ(BB) → σ*(NN) (two sets, *E*^(2)^ = −5.8 kcal/mol for each), and σ(BH) → σ*(NN) (two sets, *E*^(2)^ = −7.5 kcal/mol for each) (Figure 10a). Conjugative interactions between the LP(N) and π*(BB) bonds also stabilize the B center: LP(N) → π*(BB) (two sets, *E*^(2)^ = −11.1 kcal/mol for each) (Figure 10b). Here, “two sets” means that there are other sets of equivalent interactions by symmetry.

Among the [(N_5_)_2_B_2_X_2_]^2–^ species, the [(N_5_)_2_B_2_H_2_]^2–^ compound has the highest *E*_TS_ and the most negative boron atomic charge (*q*_*B*_ in Table 3), indicating that electron delocalization effectively quenches positive charge at the electron deficient boron center. The surprisingly low NPA charge on the B atom for the X = H species is readily noticed and suggests a highly stabilized boron center.

## 4 Conclusions

In summary, the [(N_5_)_2_BX]^2–^ and [(N_5_)_2_B_2_X_2_]^2–^ (X = H, F, Cl, and Br) compounds all exhibit both thermodynamic and kinetic stabilities, indicating possible potential catalytic agents.

The natural bond orbital (NBO), localized orbital locator (LOL) analyses, and electron localization function (ELF) analyses reveal that both π-conjugation and σ-hyperconjugation effects can effectively stabilize the [(N_5_)_2_BX]^2–^ and [(N_5_)_2_B_2_X_2_]^2–^ (X = H, F, Cl, and Br) compounds. Based on their delocalizaton energies, the (hyper)conjugative effects on the B center in these substituted [(N_5_)_2_BX]^2–^ and [(N_5_)_2_B_2_X_2_]^2–^ structures follow the order of F < Cl < Br < H. This is of course opposite to the order of their electronegativities. Due to the high electronegativities of the halogens, electron withdrawal leads to system less stability than hydrogen. Our findings suggest that [(N_5_)_2_BH]^2–^ and [(N_5_)_2_B_2_H_2_]^2–^ are possible synthetic targets of the novel borinium anions.
